# Appraising circular RNAs as novel biomarkers for the diagnosis and prognosis of gastric cancer: A pair‐wise meta‐analysis

**DOI:** 10.1002/jcla.23303

**Published:** 2020-03-20

**Authors:** Hongjun Chen, Kun Wang, Dongxu Pei, Haisheng Xu

**Affiliations:** ^1^ Department of Clinical Laboratory Anyang Tumor Hospital The Fourth Affiliated Hospital of Henan University of Science and Technology Anyang China; ^2^ Department of Clinical Laboratory Huanghe Sanmenxia Hospital Sanmenxia China; ^3^ Department of Clinical Laboratory Henan Province Hospital of TCM Zhengzhou China

**Keywords:** circular RNA, clinicopathologic feature, diagnoses, gastric cancer, prognoses

## Abstract

**Background:**

Circular RNAs (circRNAs), proven as single‐stranded closed RNA molecules, have been implicated in the onset and development of multiple cancers. This study aimed to summarize existing evidences regarding the clinicopathologic, diagnostic, and prognostic significances of circRNAs in gastric cancer (GC).

**Methods:**

Eligible studies were identified using online databases. The quality of the included studies was judged, and patients' clinical characteristics, diagnostic data, and overall survival (OS) were extracted from the electronic medical record. Fisher's method was adopted to determine *P* values for clinicopathologic features. The diagnostic and prognostic data from all included studies were merged.

**Results:**

Thirty eligible studies were comprised of 2687 GC patients were enrolled in the meta‐analyses. Altered expressions of circRNAs in GC tissues were significantly associated with worse clinicopathologic features. Abnormally expressed circRNAs yielded a pooled sensitivity of 0.76 (95% CI: 0.69‐0.81) and a specificity of 0.77 (95% CI: 0.70‐0.83) in distinguishing GC from noncancerous controls, which corresponded to an area under the curve (AUC) of 0.83. The survival analysis showed that the oncogenic circRNA signature could be an independent risk factor of OS (HR = 2.11, 95% CI: 1.60‐2.78, *P* = .000). Patients with down‐regulated circRNAs (tumor suppressor genes) presented a significantly shorter OS time than those with high‐level circRNAs (HR = 0.33, 95% CI: 0.27‐0.42, *P* = .000). Stratified analyses based on sample type, control source, circRNA expression status, and cutoff setting also produced robust results.

**Conclusions:**

CircRNAs may play an important role as potential diagnostic and prognostic biomarkers of GC.

## 1 INTRODUCTION

Gastric cancer (GC) is a major aggressive malignancy of the digestive system and a leading cause of cancer deaths across the world.^1^ Over the past three decades, the incidence rate of GC has climbed rapidly, placing considerable economic burden on healthcare systems globally.^2^ Although therapeutic technologies for GC have been vastly upgraded in recent years, the 5‐year survival rate of patients with GC, particularly advanced stage GC, still remains relatively low.^3^ As such, early diagnosis and selection of high‐risk individuals with poor prognosis are the preoccupation for achieving successful clinical research results. Endoscopy followed by pathological analysis is commonly known as the gold standard for diagnosing GC. However, many patients decline gastroscopy due to the invasive nature of the technique. The sensitivity and specificity of currently used blood biomarkers for GC detection such as carcinoembryonic antigen (CEA), carbohydrate antigen 19‐9 (CA19‐9), and carbohydrate antigen (CA72‐4) are unfavorable.^4^ Furthermore, no suitable markers for monitoring the prognosis have yet been identified. So it is the first imperative to screen out novel effective biomarkers for GC to aid early diagnosis and guide treatment planning.

Among thousands of predicted tumor biomarkers for cancers, circRNAs are a special group of endogenous coding/non‐coding RNAs with a complete ring structure formed by jointing 3′ and 5′ ends together via exon or intron circularization.^5^ As previously reported, circRNAs participate in multiple physiological activities,^6^ while their dysregulation involves in the pathogenesis of cancers.^7^ Likewise, dysregulated circRNAs as significant clinicopathologic, diagnostic, and/or prognostic factors for GC have been extensively investigated so far.^8^, ^9^, ^10^, ^11^, ^12^, ^13^, ^14^, ^15^, ^16^, ^17^, ^18^, ^19^, ^20^, ^21^, ^22^, ^23^, ^24^, ^25^, ^26^, ^27^, ^28^, ^29^, ^30^, ^31^, ^32^, ^33^, ^34^, ^35^, ^36^, ^37^, ^38^, ^39^ However, such use in daily clinical practice has not been approved. So the aim of the current meta‐analysis was to retrieve original studies that assessed their associations with clinicopathologic features and diagnostic and prognostic potential of GC.

## 2 MATERIALS AND METHODS

### 2.1 Study selection

A wide range of databases encompassing PubMed, Embase, Web of Science, EBSCO, BioMed Central, and CNKI were searched for eligible studies indexed until March 1, 2019. Search terms were combined with “AND/OR” and were listed as follows: “gastric cancer”, “GC”, “gastric carcinoma”, “stomach cancer”, “cancer of the stomach”, “circular RNA”, “circRNA”, “hsa circ”, “clinicopathologic features”, “clinicopathological characteristics”, “clinicopathological parameters”, “clinical and pathological characteristics”, “clinical pathologic characteristics”, “diagnosis”, “diagnoses”, “sensitivity”, “specificity”, “ROC curve”, “AUC”, “area under the curve”, “prognosis”, “prognoses”, “HR”, “hazard ratio”, “overall survival”, “OS”, “disease‐free survival”, “DFS”, “EFS”, “event‐free survival”, “progression‐free survival”, and “PFS”. The associated reference lists included in each study were also manually searched to increase search sensitivity.

### 2.2 Selection standards

Inclusion criteria were defined as follows: (a) Studies were limited to those that assessed the diagnostic and/or prognostic value of circRNA(s) in patients with GC; (b) all patients were definitely diagnosed as GC with pathological evidence and did not receive any preoperative clinical treatments prior to sampling; (c) for diagnostic studies, the numerical values for true positive (TP), false positive (FP), false negative (FN), and true negative (TN) were available or could be calculated indirectly; and (d) studies provided an estimate of HR(s) and associated 95% CIs for prognosis, or these values could be calculated indirectly based on the Kaplan‐Meier survival curves. Exclusion criteria were as follows: (a) studies on cancers other than GC; (b) studies with insufficient data for statistical analysis or that were rated as low quality; (c) studies with full texts not completely written in English; or (d) research data based on basic science experiments, or animal samples, or case reports, reviews, comments, and letters.

### 2.3 Data extraction

Two authors independently retrieved the name of the first author, year of publication, country, study design, case numbers, sample types, control sources, circRNA signatures, test methods, cutoff value settings, reference genes, values of sensitivity and specificity, HR values with 95% CIs, and follow‐up time. Any disagreement was resolved by group discussion until consensus was reached.

### 2.4 Study bias and quality assessment

We first used the Quality Assessment for Studies of Diagnostic Accuracy 2 (QUADAS‐2) checklist to judge the quality and bias of the eligible studies that evaluated diagnostic performances of circRNA(s) in GC.^40^ The QUADAS‐2 checklist was composed of two parts, “risk of bias” and “applicability concerns,” and contained seven items categorized into patient selection, index test, reference standard, flow, and timing. Each item could be rated as low risk, high risk, or unclear risk, and an answer of “low risk” merely received 1 point, while that of either “high risk” or “unclear risk” did not receive any point. In addition, guidelines from the Newcastle‐Ottawa Quality Assessment Scale (NOS) checklist were used to determine the bias of prognostic studies,^41^ in which eight items regarding study selection, comparability, and outcome were addressed. Risk of bias was judged as low risk, high risk, or unclear risk, corresponding to quantitative scores of 1, 0, and 0 points.

### 2.5 Statistical analysis

Statistical analyses were conducted using STATA (version 12.0) and Meta‐DiSc software (version 1.4). The estimated *I* ^2^ and *Chi‐square* statistics were used to assess the heterogeneity among studies. A *P‐*value of <0.1 in the *Chi‐square* test with *I* ^2^ of >50% indicated significant heterogeneity. Fisher's method was used to combine the *P* values for clinicopathologic features. Pooled estimates of sensitivity, specificity, positive likelihood ratio (PLR), negative likelihood ratio (NRL), diagnostic odds ratio (DOR), and HRs with 95% CIs were calculated using a random effect model when significant heterogeneity was observed. Otherwise, a fixed‐effect model was used. Influence and meta‐regression tests were performed to trace the underlying causes of study heterogeneity. Deek's funnel plot, and Begg's and Egger's tests were adopted to analyze qualitative publication bias, and a *P‐*value of <.05 was considered statistically significant. When publication bias was observed, the trim‐and‐fill method was used to assess the possible effects of bias on the overall pooled effects.^42^

## 3 RESULTS

### 3.1 Search results and study characteristics

**Figure 1:**
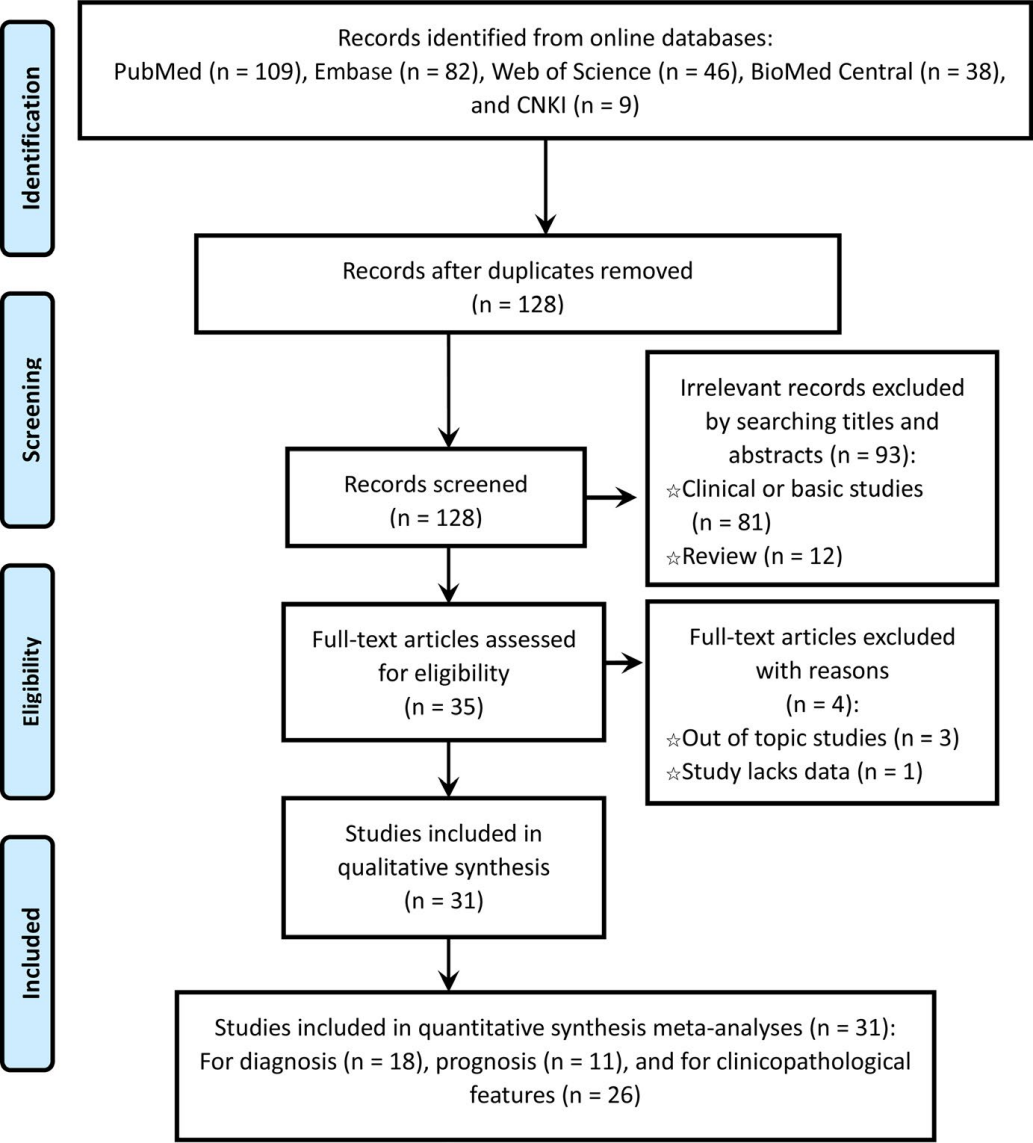
The flow diagram of the study selection procedure

As summarized in Figure 1, 128 studies were obtained by searching 6 weeks databases. Then, we scanned the titles and abstracts of these manuscripts and removed 93 articles because the topics were not within the scope of this study. Thirty studies^8^, ^9^, ^10^, ^11^, ^12^, ^13^, ^14^, ^15^, ^16^, ^17^, ^18^, ^19^, ^20^, ^21^, ^22^, ^23^, ^24^, ^25^, ^26^, ^27^, ^28^, ^29^, ^30^, ^31^, ^32^, ^33^, ^34^, ^35^, ^36^, ^37^ including 21 studies on clinicopathologic features,^8^, ^9^, ^10^, ^11^, ^12^, ^13^, ^14^, ^15^, ^16^, ^17^, ^23^, ^24^, ^26^, ^27^, ^28^, ^29^, ^30^, ^33^, ^35^, ^36^, ^37^ 19 on diagnosis,^8^, ^9^, ^10^, ^11^, ^12^, ^13^, ^14^, ^15^, ^16^, ^17^, ^18^, ^23^, ^24^, ^27^, ^28^, ^29^, ^31^, ^34^, ^37^ and 11 on prognosis^19^, ^20^, ^21^, ^22^, ^23^, ^25^, ^27^, ^32^, ^33^, ^34^, ^35^ were included in the meta‐analysis.

All essential data were obtained from the 30 studies (Tables 1, 2, 3), representing 2687 GC cases composed of 1566 who tested circRNAs for clinicopathologic features, 1462 for diagnosis, and 1167 for prognosis. All studies were conducted among Asian populations comprising a large group of Chinese cases. All GC patients were diagnosed pathologically, and specimens (tissue or plasma) were obtained prior to any clinical treatment. A circRNA signature contained 33 circRNAs, of which 15 showed oncogenic functions featuring up‐regulations in GC and the rest were tumor suppressor genes. Targeted circRNA levels were measured by quantitative reverse transcription‐polymerase chain reaction (qRT‐PCR), or RNA‐seq analyses, and were normalized to *GAPDH*, *β‐actin*, or *U*6 mRNAs. The control sources consisted of paired adjacent noncancerous tissues or biopsies from healthy individuals. Among the 11 studies over circRNAs and prognosis of GC, 6 directly reported HRs and 5 showed survival curves from which HRs could be calculated. The survival point only included OS, and the datasets for DFS and RFS were eliminated from our analysis due to limited study size. ^38^, ^39^

**Table 1:** The individual *P* values of the included studies which assessed the associations between circRNA levels and clinicopathologic features

Study	Sex	Age	Diameter	Differentiation grade	T stage	Distant metastasis	TNM stage	Lymphatic metastasis	Venous invasion	Nervous invasion	AFP	CEA	CA199	CA724
Chen J 2017^19^	0.138	0.551	0.174	0.188	0.02	0.494	0.194	0.464	/	0.03	/	/	/	/
Pan H 2017^20^	/	/	/	/	/	0.0205	/	/	/	/	/	/	/	/
Zhang Y 2017^21^	0.794	0.141	/	0.019	/	/	0.415	0.03	/	/	/	/	/	/
Zhang J 2017^22^	/	/	/	/	/	/	/	/	/	/	/	/	/	/
Rong D 2019^23^	0.25	0.53	0.266	0.309	/	/	0	0.021	/	/	/	/	/	/
Sun H 2018^24^	0.064	0.491	0.55	0.811	/	/	0.002	0.744	/	/	0.284	0.624	/	/
Cheng J 2018^26^	0.807	0.706	0.174	0.49	0.004	0.494	/	0.55	/	/	/	/	/	/
Sun H 2018^27^	0.553	0.545	0.588	0.189	/	/	0.026	0.12	/	/	0.222	0.351	0.455	0.603
Rong D 2018^28^	0.083	0.087	0.454	/	/	/	0.262	0.023	/	/	/	0.207	0.375	/
Huang M 2017^29^	0.203	0.757	0.168	0.012	/	/	0.056	0.064	/	/	/	0.077	/	/
Ghasemi S 2019^30^	0.5	0.01	0.5	/	0.5		0.31	0.32	/	0.5	/	/	/	/
	0.36	0.005	0.5	/	0.5	/	0.31	0.34	/	0.35	/	/	/	/
Li X 2019^33^	0.793	0.599	/	0.144	0.028	/	0.014	0.279	/	/	/	/	/	/
Lu J 2018^34^	0.418	0.136	0.353	0.145	0.001	/	0.001	0.001	/	/	/	0.752	/	0.561
Chen Y^35^	/	/	/	0.031	/	/	0.002	/	/	/	/	/	/	/
Xu Y 2018^36^	0.82	0.483	0.035	0.008	/	/	0.213	0.221	/	/	/	/	/	/
Xie Y 2018^37^	0.815	0.355	0.574	0.116	0.333	0.261	0.361	0.039	/	/	/	0.058	0.027	/
Chen S 2017^8^	0.17	0.835	0.034	0.904	/	0.001	/	0.026	/	/	/	0.303	0.019	/
Li P 2015^9^	0.002	0.022	0.229	0.698	0.264	0.036	0.042	0.429	/	/	/	0.541	0.871	/
Li WH 2017^10^	0.834	0.549	/	0.039	0.366	/	0.386	0.389	/	/	/	0.914	0.958	0.118
Lu R 2017^11^	0.815	0.327	0.761	0.235	0.492	0.037	/	0.224	0.519	0.284	/	0.041	0.147	/
Shao Y 2017^12^	0.326	0.746	0.27	0.77	/	0.917	0.516	0.571	0.655	0.507	/	0.345	0.01	/
Shao Y 2017^13^	0.524	0.84	0.74	0.042	0.431	0.74	/	0.698	0.683	0.753	/	0.001	0.097	/
Shao Y 2017^14^	0.398	0.727	0.706	0.24	0.123	0.048		0.768	0.329	0.062	/	0.001	0.021	/
Sun H 2017^15^	0.663	0.29	0.185	0.355			0.03	0.254	/	/	0.293	0.535	/	/
Tian M 2017^16^	0.003	0.657	0.095	0.915	0.116	0.02	0.018	0.325	/	/	/	0.921	0.031	/
Zhao Q 2018^17^	0.362	0.71	0.027	0.673	0.743	0.023	0.1	0.044	/	/	/	/	/	/
*Chi* ^2^ value	65.51	60.50	59.20	79.36	61.70	62.56	130.05	93.13	5.14	20.50	7.98	58.	51.	6.44
Pooled *P*	.0470	.1060	.0410	.0009	.0000	.00003	.0000	.00010231	.7420955	.11504307	.24	.0012	.0004	.38

**Table 2:** Characteristics of the included diagnostic studies that evaluated circRNAs in GC

Author	Year	Country	Control type	Test matrix	Method	Cutoff value	Control gene	CircRNA signature	Expression	GC size	Control size	AUC	QUADAS score
Lu R^11^	2017	Chinese	PANS	Tissue	qRT‐PCR	8.17	*GAPDH*	Hsa_circ_0006633	Decreased	96	96	0.741	6
Zhao Q^17^	2018	Chinese	PANS	Tissue	qRT‐PCR	9.40	*GAPDH*	Hsa_circ_0000181	Decreased	115	115	0.756	6
			HS	Plasma	qRT‐PCR	7.27	*GAPDH*	Hsa_circ_0000181	Decreased	102	105	0.582	
Xie Y^37^	2018	Chinese	PANS	Tissue	qRT‐PCR	12.17	*GAPDH*	Hsa circ 0 074 362	Decreased	127	127	0.630	6
Huang M^29^	2017	Chinese	PANS	Plasma	qRT‐PCR	Unclear	*GAPDH*	Hsa_circ_0000745	Decreased	60	60	0.683	5
Li P^9^	2015	Chinese	PANS	Tissue	qRT‐PCR	12.9	*GAPDH*	Hsa_circ_002059	Decreased	101	101	0.730	5
Chen S^8^	2017	Chinese	PANS	Tissue	qRT‐PCR	6.83	*GAPDH*	Hsa_circ_0000190	Decreased	104	104	0.750	5
Sun H^15^	2018	Chinese	PANS	Tissue	qRT‐PCR	Unclear	*GAPDH*	Hsa_circ_0000520	Decreased	56	56	0.613	4
			HS	Plasma	qRT‐PCR	Unclear	*GAPDH*	Hsa_circ_0000520	Decreased	45	17	0.897	4
Shao Y^13^	2017	Chinese	PANS	Tissue	qRT‐PCR	9.53	*GAPDH*	Hsa_circ_0001895	Decreased	96	96	0.792	6
Tian M^16^	2018	Chinese	PANS	Tissue	qRT‐PCR	12.31	*GAPDH*	Hsa_circ_0003159	Decreased	108	108	0.750	6
Shao Y^12^	2017	Chinese	PANS	Tissue	qRT‐PCR	9.125	*GAPDH*	Hsa_circ_0000705	Decreased	96	96	0.719	6
		Chinese	PANS	Tissue	qRT‐PCR	12.14	*GAPDH*	Hsa_circ_0014717	Decreased	96	96	0.696	6
Li WH^10^	2017	Chinese	PANS	Tissue	qRT‐PCR	0.226923	*GAPDH*	Hsa_circ_00001649	Decreased	76	76	0.834	6
Lai Z^18^	2017	Chinese	PANS	Tissue	qRT‐PCR	Unclear	*β‐actin*	CircRNA0047905	Increased	31	31	0.850	4
			PANS	Tissue	qRT‐PCR	Unclear	*β‐actin*	CircRNA0138960	Increased	31	31	0.647	4
			PANS	Tissue	qRT‐PCR	Unclear	*β‐actin*	CircRNA7690‐15	Increased	31	31	0.681	4
Rong D^23^	2019	Chinese	PANS	Tissue	qRT‐PCR	9.965	*GAPDH*	CircPSMC3	Decreased	106	106	0.9326	6
Sun H^24^	2018	Chinese	PANS	Tissue	qRT‐PCR	−11.46	*GAPDH*	Circ‐sFMBT2	Increased	36	36	0.7585	5
Sun H^27^	2018	Chinese	PANS	Tissue	qRT‐PCR	Unclear	*GAPDH*	CircPVRL3	Decreased	62	62	0.7626	4
Rong D^28^	2018	Chinese	PANS	Tissue	qRT‐PCR	Unclear	*GAPDH*	Circ_0066444	Increased	106	106	0.7328	6
Li T^31^	2018	Chinese	HS	Plasma	qRT‐PCR	Unclear	*GAPDH*	Hsa_circ_0001017	Decreased	121	121	0.849	5
			HS	Tissue	qRT‐PCR	Unclear	*GAPDH*	Hsa_circ_0001017	Decreased	121	121	0.732	
			HS	Plasma	qRT‐PCR	Unclear	*GAPDH*	Hsa_circ_0061276	Decreased	121	121	0.851	5
			HS	Tissue	qRT‐PCR	Unclear	*GAPDH*	Hsa_circ_0061276	Decreased	121	121	0.78	
			HS	Plasma + tissue	qRT‐PCR	Unclear	*GAPDH*	Hsa_circ_0001017	Decreased	242	242	0.868	
			HS	Plasma + tissue	qRT‐PCR	Unclear	*GAPDH*	Hsa_circ_0061276	Decreased	242	242	0.952	
Lu J^32^	2018	Chinese	HS	Plasma	qRT‐PCR	Unclear	*GAPDH*	Hsa_circ_0000467	Increased	20	20	0.79	5

Abbreviations: AUC, area under the curve; GAPDH, glyceraldehyde‐3‐phosphate dehydrogenase; GC, gastric cancer; HS: healthy sample; PANS, paired adjacent noncancerous sample; QUADAS, Quality Assessment for Studies of Diagnostic Accuracy 2.

**Table 3:** Characteristics of the included prognostic studies that evaluated circRNAs in GC

Author	Year	Case	Sample type	Method	Control gene	circRNA signature	Cutoff High/low	Outcome	*P* value	Follow‐up (mon)	HR extraction	NOS score
Chen J^19^	2017	187	Tissue	RNA‐seq analyses	/	circPVT1	107/80	OS	.008	Median:26	Directly	8
	2017	187	Tissue	RNA‐seq analyses	/	circPVT1	107/80	OS	.047	Median:26	Directly	
Pan H^20^	2017	102	Tissue	qRT‐PCR	U6	ciRS‐7	50/52	OS	.0143	Unclear	Directly	6
		154	Tissue	qRT‐PCR	U6	ciRS‐7	83/71	OS	.0061	Unclear	Directly	6
Zhang Y^21^	2017	112	Tissue	qRT‐PCR	Unclear	circRNA_100269	28/64	OS	.02	Unclear	Directly	6
Zhang J^22^	2017	80	Tissue	qRT‐PCR	*GAPDH*	circLARP4	41/39	OS	.002	Unclear	Directly	6
		80	Tissue	qRT‐PCR	*GAPDH*	circLARP4	41/39	OS	.036	Unclear	Directly	6
Rong D^23^	2019	106	Tissue	qRT‐PCR	*GAPDH*	circPSMC3	15/91	OS	.0022	Unclear	Indirectly	6
Liu H^25^	2018	80	Tissue	qRT‐PCR	*GAPDH*	circYAP1	43/37	OS	.0061	Unclear	Indirectly	6
	2018	42	Tissue	qRT‐PCR	*GAPDH*	circYAP1	20/22	OS	.0405	Unclear	Indirectly	6
Sun H^27^	2018	62	Tissue	qRT‐PCR	*GAPDH*	CircPVRL3	15/47	OS	.007	Unclear	Directly	6
		32	Tissue	qRT‐PCR	*GAPDH*	CircPVRL3	4/28	OS	.039	Unclear	Directly	6
Lu J^32^	2019	20	Tissue	qRT‐PCR	*GAPDH*	hsa_circ_0001368	NR	OS	Unclear	Unclear	Indirectly	5
Li X^33^	2019	58	Tissue	qRT‐PCR	*Unclear*	circ‐ERBB2	29/29	OS	.022	Unclear	Indirectly	6
Lu J^34^	2018	51	Tissue	qRT‐PCR	*GAPDH*	hsa_circ_0000467	32/19	OS	.032	Median: 32	Directly	8
		51	Tissue	qRT‐PCR	*GAPDH*	hsa_circ_0000467	32/19	OS	.041	Median: 32	Directly	
Chen Y^35^	2018	81	Tissue	qRT‐PCR	*GAPDH*	circAGO2	40/41	OS	.0001	Unclear	Indirectly	6

Abbreviations: GAPDH, glyceraldehyde‐3‐phosphate dehydrogenase; HR, hazard ratio; NOS, Newcastle‐Ottawa Quality Assessment Scale; OS, overall survival.

### 3.2 Quality assessment

For diagnostic effects, studies were rated for patient selection, index test, reference standard, flow, and timing by the QUADAS‐II criteria with a maximum of seven points.^40^ As shown in Table S1, all studies received rated QUADAS scores of ≥4 points. Prognostic studies were assessed using the NOS checklist with a maximum of nine points,^41^ and all 11 studies achieved NOS scores of ≥ 6 (Table S2). The results suggested that risks of bias and quality in the studies were acceptable.

### 3.3 CircRNA expressions and clinicopathologic features

As shown in Table 1, altered circRNA levels in tissues of GC patients were significantly associated with gender (*P* = .0470), tumor lesion diameter (*P* = .0410), differentiation grade (*P* = .0009), T stage (*P* = .0003), distant metastasis (*P* = .0000), TNM stage (*P* = .0000), lymphatic metastasis (*P* = .0001), CEA (*P* = .0012), and CA199 levels (*P* = .0004). Other clinicopathologic factors such as age, venous invasion, nervous invasion, AFP, and CA724 merely showed no associations with circRNA expressions (Table 1).

### 3.4 Diagnostic performance

**Figure 2:**
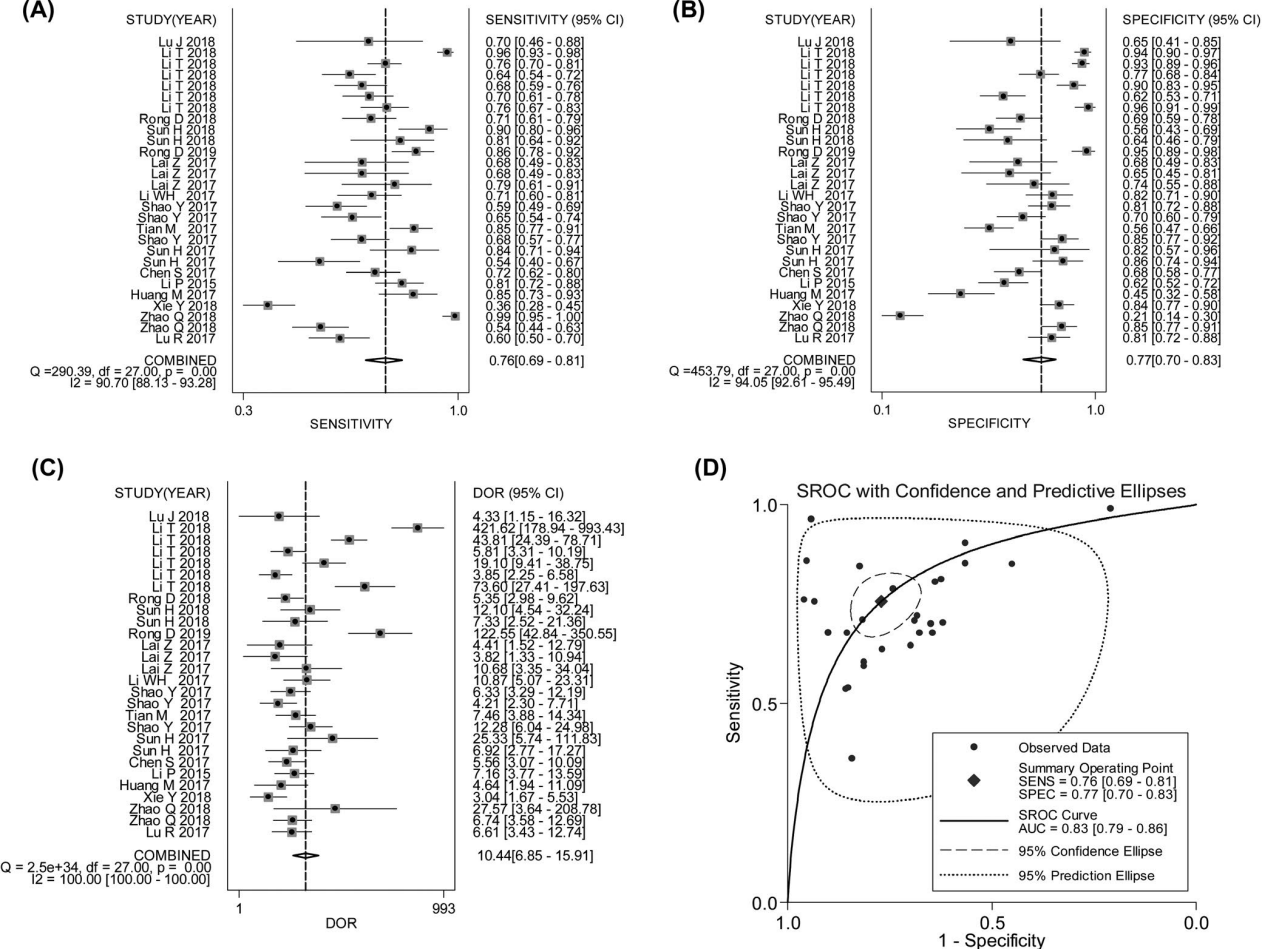
Forest plots of pooled sensitivity (A), specificity (B), DOR (C), and SROC curve (D) for circRNAs in diagnosing GC

The area under the SROC curve of circRNAs for distinguishing GC from noncancerous controls was 0.83 (heterogeneity: *I* ^2^ = 99.43%; Q = 353.467, *df* = 2.00, *P* = .000), with pooled sensitivity of 0.76 (95% CI: 0.69‐0.81), specificity of 0.77 (95% CI: 0.70‐0.83), and DOR of 10.44 (95% CI: 6.85‐15.91) (Figure 2). The combined PLR and NLR were estimated at 3.30 (95% CI: 2.51‐4.34) and 0.32 (95% CI: 0.25‐0.40), respectively.

The diagnostic efficacy of circRNAs for GC was further determined in terms of test matrix, control source, cutoff setting, and circRNA expression status. As summarized in Table 4, the results showed that plasma circRNA tests achieved greater accuracy than tissue circRNA test, with an AUC of 0.87 and DOR of 16.00. Furthermore, we compared the efficacy of circRNA expression signature in distinguishing GC and noncancerous controls. Our data demonstrated that circRNA expression as a diagnostic tool is more prominent in differentiating GC patients from healthy individuals than in distinguishing GC from paired adjacent noncancerous controls (AUC: 0.90 vs 0.79; DOR: 22.79 vs 7.18; sensitivity: 0.80 vs 0.69; specificity: 0.81 vs 0.74). In addition, a comparison of circRNA expression status showed that the AUC (0.85 vs 0.74) and the DOR (12.22 vs 5.50) of down‐regulated circRNA (tumor suppressor genes) expressions were higher than those of up‐regulated circRNAs (oncogenes). Finally, diagnostic accuracy was dependent on cutoff settings: a cutoff value setting of <10 yielded higher efficacy than that of ≥10 (AUC: 0.83 vs 0.77; DOR: 10.13 vs 5.58).

**Table 4:** The stratified analysis of the pooled efficacy of circRNAs for the diagnosis of GC

Analyses	*I* ^2^/ *P* value (Chi^2^)	Sensitivity (95% CI)	Specificity (95% CI)	PLR (95% CI)	NLR (95% CI)	DOR (95% CI)	AUC
Test matrix							
Plasma	77%/*P* = .0006	0.81 (0.77‐0.84)	0.68 (0.63‐0.72)	3.51 (1.28‐9.64)	0.29 (0.21‐0.40)	16.00 (6.20‐41.26)	0.87
Tissue	63.6%/*P* = .0001	0.68 (0.65‐0.70)	0.75 (0.72‐0.77)	2.63 (2.26‐3.07)	0.41 (0.35‐0.49)	6.94 (5.30‐9.07)	0.79
Control type							
GC vs Paired adjacent noncancerous tissue	62.8%/*P* = .0001	0.69 (0.66‐0.71)	0.74 (0.72‐0.76)	2.62 (2.20‐3.13)	0.40 (0.33‐0.48)	7.18 (5.39‐9.56)	0.79
GC vs Healthy individual	93.4%/*P* = .0000	0.80 (0.77‐0.82)	0.81 (0.78‐0.83)	4.78 (1.65‐13.91)	0.25 (0.17‐0.38)	22.79 (7.91‐65.67)	0.90
Expression status							
Up‐regulated circRNAs	0%/*P* = .807	0.72 (0.66‐0.78)	0.68 (0.62‐0.74)	2.23 (1.84‐2.71)	0.42 (0.33‐0.51)	5.50 (3.76‐8.06)	0.74
Down‐regulated circRNAs	89.3%/*P* = .000	0.73 (0.72‐0.75)	0.78 (0.76‐0.80)	3.63 (2.50‐5.26)	0.33 (0.26‐0.42)	12.22 (7.58‐19.69)	0.85
Cutoff setting							
Cutoff value ≥ 10	44.8%/*P* = .1427	0.64 (0.59‐0.69)	0.72 (0.67‐0.76)	2.21 (1.85‐2.64)	0.43 (0.25‐0.74)	5.58 (3.64‐8.57)	0.77
Cutoff value <10	77.9%/*P* = .000	0.72 (0.69‐0.75)	0.73 (0.70‐0.76)	3.23 (1.72‐6.08)	0.37 (0.28‐0.48)	10.13 (5.81‐17.67)	0.83

Abbreviations: AUC, area under the curve; DOR, diagnostic odds ratio; GC, gastric cancer; NRL, negative likelihood ratio; PLR, positive likelihood ratio.

### 3.5 Prognostic value

**Figure 3:**
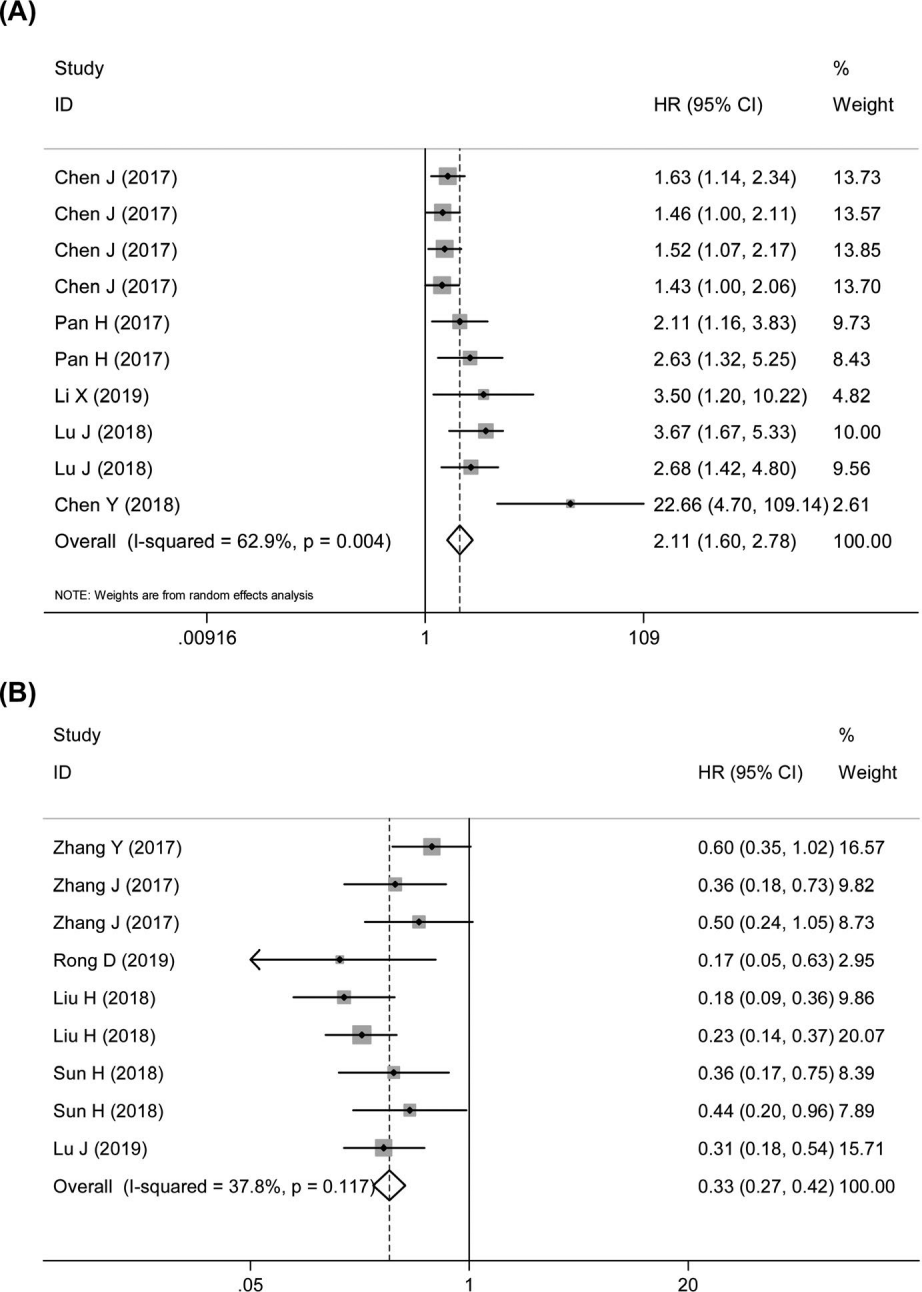
Forest plots of pooled HRs with 95% CIs of oncogenic circRNAs (A) and tumor suppressor circRNAs (B) for predicting OS of GC patients

The prognostic ability of circRNA expression status was evaluated. Multivariate Cox hazard regression analysis indicated that GC patients featuring increased oncogenic circRNA expressions had a worse OS than those with low circRNA levels (HR = 2.11, 95% CI: 1.60‐2.78, *P* = .000; heterogeneity: *I* ^2^ = 62.9%, *P* = .004) (Figure 3A). In addition, highly expressed circRNAs acting as tumor suppressors indicated favorable prognoses in GC patients (HR = 0.33, 95% CI: 0.27‐0.42, *P* = .000; heterogeneity: *I* ^2^ = 37.8%, *P* = .117) (Figure 3B).

### 3.6 Influence and meta‐regression tests

**Figure 4:**
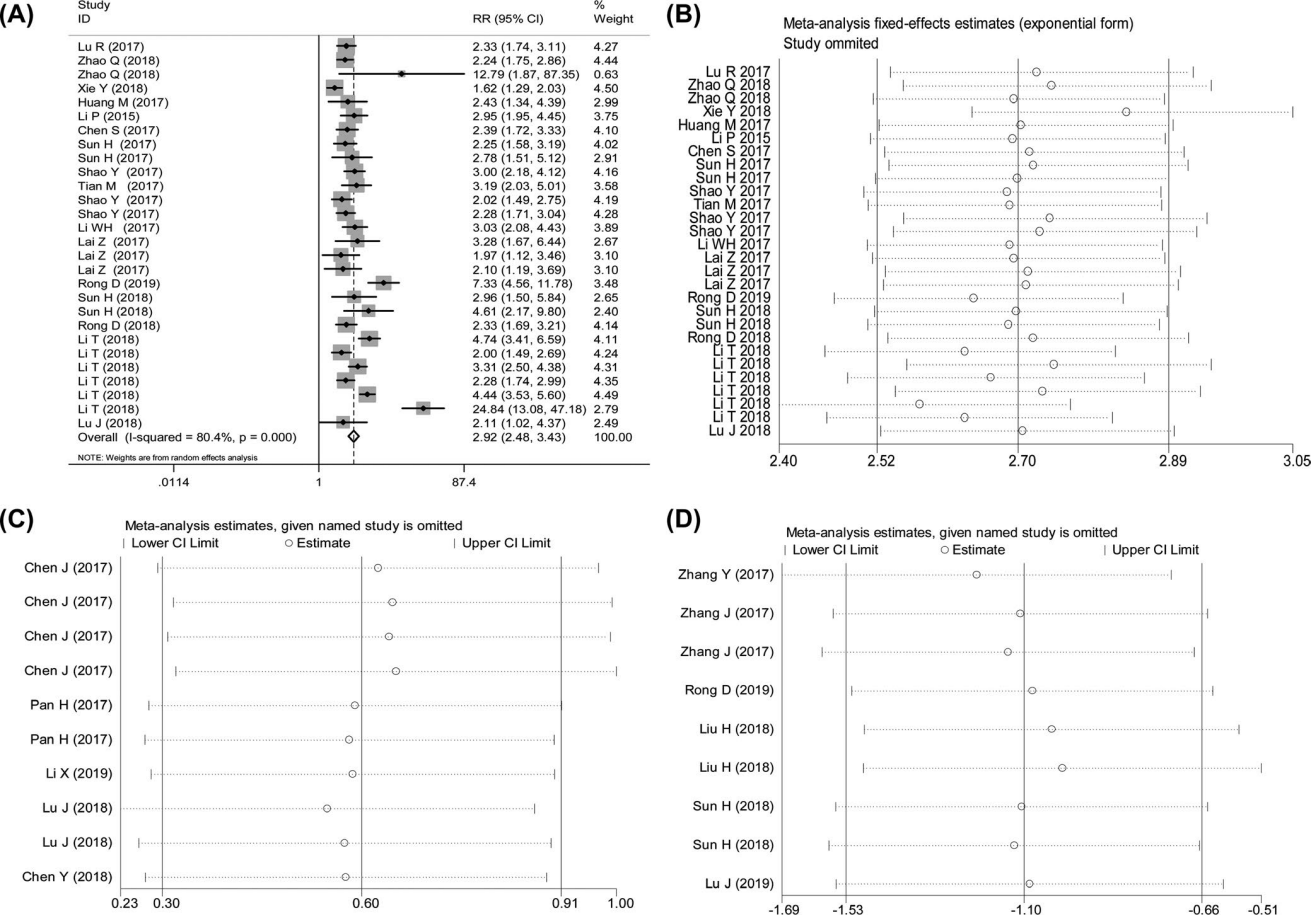
The sensitivity analysis of data homogeneity for the pooled diagnostic and prognostic effects (A, B) of oncogenic circRNAs (C) and tumor suppressor circRNAs (D)

The sensitivity test showed that all studies with available analyses for the diagnostic and prognostic effects of circRNAs were equally distributed within the lower and upper limits of the 95% CI, and no individual outlier studies were included (Figure 4).

Meta‐regression tests were conducted for control type, test matrix, cutoff setting, expression status, sample size, and QUADAS score. The results showed that different test matrices contributed to the significant heterogeneity observed in this study, with a *P* value of .0001 and PDOR of 3.46 (95% CI: 2.01‐5.94). Other covariates did not significantly contribute to heterogeneity (data not shown in full).

### 3.7 Publication bias

**Figure 5:**
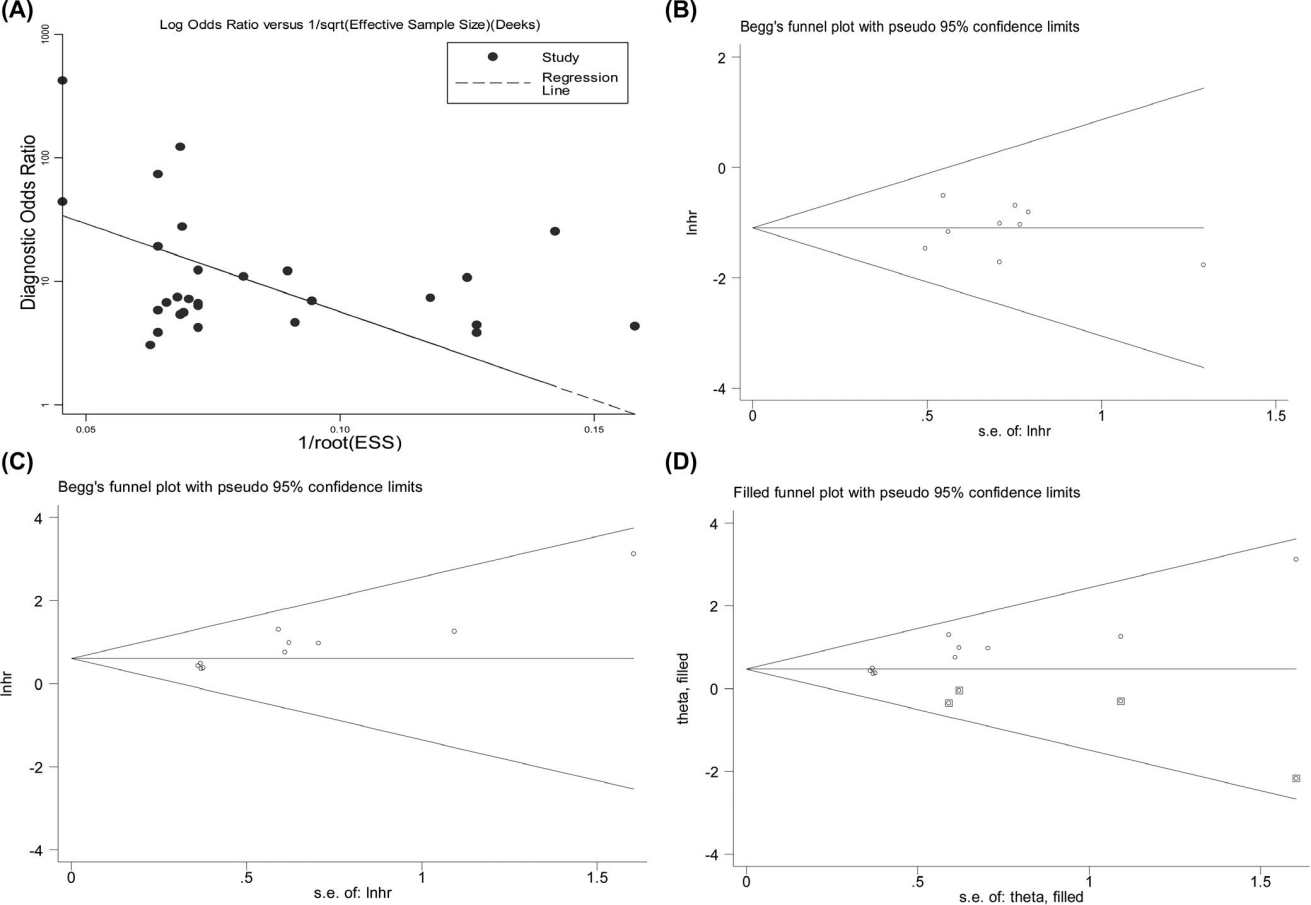
The assessment of publication bias among studies. A, bias in diagnostic effects as determined by Deek's funnel plot (*P* = .053); B, bias in the prognostic effects of tumor suppressor circRNAs as determined by Begg's test; C, Begg's funnel plot showed significant publication bias in prognostic effects of oncogenic circRNAs; D, the adjustment effect with a fixed‐effect model using the trim‐and‐fill method. A hollow circle in square represents the imputed missing studies due to negative publications

No publication bias in the pooled diagnostic effects was determined by Deek's funnel plot (*P* = .053), neither was the bias in the prognostic effects of down‐regulated circRNAs on OS by Begg's and Egger's tests (Egger's test, *P* = .806; Begg's test, *P* > .05). However, significant bias was observed in the prognostic meta‐analysis of oncogenic circRNAs for OS (Egger's test, *P* = .000). Consequently, the trim‐and‐fill method was used to more thoroughly assess possible effects of publication bias.^42^ The fixed‐effect model identified four missing studies, and the pooled adjusted effort differed little before and after adjustment (*z* = 3.854, *P* = .000 vs *z* = 3.247, *P* = .001), suggesting that the pooled effects were not subject to bias due to unpublished negative studies. The included studies generated a symmetrical funnel plot, as shown in Figure 5 (funnel plots of Egger's test were not shown).

## 4 DISCUSSION

As GC is a highly heterogeneous disease with a high mortality rate,^1^, ^2^, ^3^ most patients are confirmed until a very late stage due to the hidden symptoms. Despite the constantly updated treatments for the disease, the 5‐year survival rate is still undesirable.^3^ Identifying informative diagnostic and prognostic biomarkers of GC early on is the first priority for better predicting tumor behavior and guiding the treatment planning. That prompts a hotspot of circRNAs as a novel class of coding/non‐coding RNAs characterized by circularization through covalent bonding of their 5' and 3' ends for cancer diagnosis.^5^, ^6^ Owing to the ring structure, circRNAs are more stable and conserved than linear RNAs, and a majority of them are highly stable in tissues and bodily fluids, as confirmed by some studies.^43^, ^44^ This unique characteristic suggests that circRNAs can be reckoned as promising noninvasive biomarkers of cancers, especially GC.^45^, ^46^, ^47^ In this study, we analyzed the associations between circRNA expressions and clinicopathologic features, and determined clinical values of circRNAs as diagnostic and prognostic indicators of GC.

We summarize the correlation between tissue circRNA expressions and the basic characteristics, and find that several major clinical features such as gender, tumor diameter, differentiation grade, T stage, distant metastasis, TNM stage, lymphatic metastasis, and CEA and CA199 levels are markedly linked to circRNAs levels (Table 1). This indicates that circRNAs involve in the onset, development, and progression of GC. Interestingly, we find gender as an independent factor associated with circRNA expressions in this analysis. Previous studies have reported that expressions of some circRNAs (eg, hsa_circ_002059, hsa_circ_0003159) in tissues are linked to gender.^9^, ^16^ The majority of the GC cases expressing the aforesaid circRNAs are over 60 years old and male patients are predominant,^1^, ^2^, ^3^ which agree with our findings. Due to limited sample size, no other correlations between circRNAs and other clinicopathological factors such as venous invasion, nervous invasion, AFP, and CA724 are observed (Table 1).

The ROC curve is a comprehensive index reflecting the efficacy of a diagnostic test. A larger AUC represents greater diagnostic value of each variable.^48^ In our diagnostic meta‐analysis, we confirm that circRNA levels are potentially valuable for the diagnosis of GC, with a combined AUC of 0.83 (Figure 2). DOR is another important index for diagnostic tests, and a higher value indicates better diagnostic efficacy.^49^ In this study, a pooled DOR of 10.44 also demonstrates that circRNA levels are a potential diagnostic indicator for distinguishing GC form noncancerous controls (Figure 2). Our findings demonstrate that circRNA expression profiling has potential as a diagnostic biomarker analysis for GC.

For the pooled diagnostic performance of circRNAs in GC, our stratified analyses of sample type, control source, circRNA function, and cutoff setting have also produced robust results. As a result, differences in the diagnostic efficacy are found to depend on test matrix, featuring that plasma circRNAs provide a better test matrix than tissue ones for the diagnosis of GC (Table 4). A previous report has proven that different sample sources can bring about disparities in the diagnostic efficacy non‐coding RNAs, which indirectly support our findings.^50^ Furthermore, our analysis has confirmed that circRNAs as a group of underlying indicators are more effective in differentiating GC patients from healthy individuals than from paired adjacent noncancerous controls (Table 4). In addition, oncogenic circRNA expressions yield better diagnostic accuracy for GC than tumor suppressor circRNAs (Table 4). Besides, it is corroborated that the cutoff value setting of <10 can result in greater efficacy than that of ≥10 (Table 4). This indicates that the diagnostic power of circRNAs in GC is sensitive to the cutoff value settings. However, no similar results have been observed in previous studies regarding control sources, circRNA functions, and cutoff settings for support of our findings, and more studies are needed.

As previously reported, some circRNAs have been found to have prognostic value in GC.^19^, ^20^, ^21^, ^22^, ^23^, ^25^, ^27^, ^32^, ^33^, ^34^, ^35^ Therefore, a meta‐analysis for the prognostic value of previously reported circRNAs in GC has been performed, and the data have been stratified into oncogenic and tumor suppressor circRNA datasets. As a result, GC patients with elevated oncogenic circRNAs merely reveal poor OS time (HR = 2.11), and increased tumor suppressor circRNA expressions are associated with a favorable OS time (HR = 0.33) (Figure 3). All this suggests that circRNAs play a significant role as biomarkers in predicting OS of GC patients. However, the analysis for predictive effects of circRNAs on DFS and RFS has not been carried out due to the dearth of eligible studies.^38^, ^39^

Heterogeneity is common when performing a meta‐analysis.^51^ However, considerable heterogeneity can be easily found in the overall diagnostic and prognostic effects of oncogenic circRNAs. To eliminate the underlying impacts of heterogeneity on the overall combined effects, we have performed a sensitivity analysis and a meta‐regression test, and the sensitivity analysis just reveals that no individual studies are outliers. This suggests that the homogeneity of our data is acceptable and the combined effects are reliable (Figure 4). In the meta‐regression test, different test matrices significantly have contributed to the heterogeneity in the diagnostic meta‐analysis. Of the included 28 individual studies in this analysis, 20 datasets have evaluated tissue and 6 plasma. It is the smaller sample size in the plasma‐based studies that may result in bias. However, we only observed publication bias in the analysis for prognostic effects of oncogenic circRNAs for OS in GC patients (Figure 5). To assess the possible effects of bias on pooled efficacy, the trim‐and‐fill method has been adopted.^42^ However, filling 4 missing studies using a fixed‐effect model has not clearly altered the effects, hinting that the pooled accuracy is not subject to publication bias.

## 5 CONCLUSIONS

In summary, circRNAs may have potential clinical significance in GC and represent promising therapeutic targets and biomarkers of GC. However, our study had some limitations including population bias, obvious heterogeneity, and diverse test matrices and controls. Further studies are necessary to confirm the results of our meta‐analysis.

## Supporting information

Table S1‐S2
