# Supplementary material for: Appraising circular RNAs as novel biomarkers for the diagnosis and prognosis of gastric cancer: A pair‐wise meta‐analysis
Source: J Clin Lab Anal. 2020 Mar 20;34(8):e23303. doi: 10.1002/jcla.23303 (PMC7439415; doi:10.1002/jcla.23303)
Supplement: Supplementary file 1 — Table S1‐S2 [file JCLA-34-e23303-s001.docx]

**Table S1.** Study quality and bias as determined by the Quality Assessment for Studies of Diagnostic Accuracy 2 (QUADAS-2) checklist.

| **Studies** | **Risk of bias** | | | |  | **Concerns regarding applicability** | | |
| --- | --- | --- | --- | --- | --- | --- | --- | --- |
|  | **A** | **B** | **C** | **D** |  | **A** | **B** | **C** |
| Lu R [11] | Unclear | Low | Low | Low |  | Low | Low | Low |
| Zhao Q [17] | Unclear | Low | Low | Low |  | Low | Low | Low |
| Xie Y [37] | Unclear | Low | Low | Low |  | Low | Low | Low |
| Huang M [29] | Unclear | Low | Low | High |  | Low | Low | Low |
| Li P [9] | Unclear | Low | Low | High |  | Low | Low | Low |
| Chen S [8] | Unclear | Low | Low | Low |  | Low | Low | Unclear |
| Sun H [15] | Unclear | Low | Low | Unclear |  | Low | Low | Unclear |
| Shao Y [13] | Unclear | Low | Low | Low |  | Low | Low | Low |
| Tian M [16] | Unclear | Low | Low | Low |  | Low | Low | Low |
| Shao Y [12] | Unclear | Low | Low | Low |  | Low | Low | Low |
| Li WH [10] | Unclear | Low | Low | Low |  | Low | Low | Low |
| Lai Z [18] | Unclear | Low | Low | Unclear |  | Low | Low | Unclear |
| Rong D [23] | Unclear | Low | Low | Low |  | Low | Low | Low |
| Sun H [24] | Unclear | Low | Low | Unclear |  | Low | Low | Unclear |
| Sun H [27] | Unclear | Low | Low | Unclear |  | Low | Low | Unclear |
| Rong D [28] | Unclear | Low | Low | Low |  | Low | Low | Low |
| Li T [31] | Unclear | Low | Low | Low |  | Low | Low | Unclear |
| Lu J [32] | Unclear | Low | Low | Unclear |  | Low | Low | Low |

| **Study** | **Cohort selection** | | | | **Comparability** | **Outcome ascertainment** | | |
| --- | --- | --- | --- | --- | --- | --- | --- | --- |
|  | **Representativeness of the exposed cohort** | **Selection of the non-exposed cohort** | **Ascertainment of exposure** | **Demonstration that outcome of interest was not present at start of study** | **Comparability of cases and controls on the basis of the design or analysis** | **Assessment of outcome** | **Was follow-up long enough for outcomes to occur** | **Adequacy of follow up of cohorts** |
| Chen J [19] | 1 | 1 | 1 | 1 | 1 | 1 | 1 | 1 |
| Pan H [20] | 1 | 1 | 1 | 1 | 1 | 1 | 0 | 0 |
| Zhang Y [21] | 1 | 1 | 1 | 1 | 1 | 1 | 0 | 0 |
| Zhang J [22] | 1 | 1 | 1 | 1 | 1 | 1 | 0 | 0 |
| Rong D [23] | 1 | 1 | 1 | 1 | 1 | 1 | 0 | 0 |
| Liu H [25] | 1 | 1 | 1 | 1 | 1 | 1 | 0 | 0 |
| Sun H [27] | 1 | 1 | 1 | 1 | 1 | 1 | 0 | 0 |
| Lu J [32] | 1 | 1 | 1 | 1 | 1 | 0 | 0 | 0 |
| Li X [33] | 1 | 1 | 1 | 1 | 1 | 1 | 0 | 0 |
| Lu J [34] | 1 | 1 | 1 | 1 | 1 | 1 | 1 | 1 |
| Chen Y [35] | 1 | 1 | 1 | 1 | 1 | 1 | 1 | 1 |

A: Patient selection; B: Index test; C: Reference standard; D: Flow and triming

**Table S2.** The quality and bias of included studies as assessed by the Newcastle-Ottawa Scale (NOS) checklist.
